# Association between *CYP2C19* polymorphism and proton pump inhibitors adverse drug reactions: a narrative review

**DOI:** 10.3389/fphar.2025.1523399

**Published:** 2025-02-12

**Authors:** Asmaa Ibrahim, Kazeem Yusuff, Ahmed Awaisu, Hazem Elewa

**Affiliations:** Department of Clinical Pharmacy and Practice, College of Pharmacy, Qatar University, Doha, Qatar

**Keywords:** proton pump inhibitors, adverse drug reactions, *CYP2C19* polymorphism, pharmacogenomics (PGx), overuse and misuse

## Abstract

Proton pump inhibitors (PPIs) are widely prescribed medications for the management of acid-related disorders, due to their effectiveness and favorable pharmacokinetics. However, the occurrence and severity of adverse drug reactions (ADRs) in patients using PPIs, particularly in relation to their association with *CYP2C19* polymorphisms, are of great concern. This association has largely been investigated through observational studies, which have shown conflicting or weak findings. Therefore, this review aims to examine the current evidence regarding the long-term ADRs of PPIs and their link to *CYP2C19* variants.

## 1 Introduction

Proton pump inhibitors (PPIs) are used by approximately 25% of adults globally (Shanika et al., 2023). This widespread use of PPIs has considerably transformed the management of acid-related diseases, due to their effectiveness, prolonged duration of action, and their superior nocturnal and postprandial pH control compared to histamine two receptor antagonists (H2RAs). PPIs act by irreversibly binding to the hydrogen-potassium ATPase (H^+^/K^+^ -ATPase) pump on the surface of parietal cells in the stomach, resulting in inhibiting gastric acid secretion (Shin and Sachs, 2008). Several PPIs have been approved by the United States Food and Drug Administration (U.S. FDA), including omeprazole, esomeprazole, lansoprazole, dexlansoprazole, pantoprazole, and rabeprazole (Strand et al., 2017).

Although the use of genetic data to predict patients’ response and medications’ safety and efficacy–known as Pharmacogenomic (PGx) – is of growing interest (Elewa and Awaisu, 2019), genetic tests related to PPIs are yet to be implemented in most clinical practices. Several studies have highlighted the effect of inter-individual variability on the response to PPIs therapy. PPIs are primarily metabolized in the liver through cytochrome P450 2C19 (CYP2C19) enzyme, and CYP3A4 to a lesser extent, leading to the formation of inactive metabolites (Dickson and Stuart, 2003). CYP2C19 is a protein enzyme encoded by *CYP2C19* - a large gene (10q23.33) located on chromosome 10. Polymorphism or allelic variation in *CYP2C19* results in different versions of the enzyme. Polymorphisms are grouped as haplotypes, which yield different enzymatic activity and can be categorized into: normal function alleles (e.g., *1), two null or no-function alleles (e.g., *2, *3), two increased function alleles (e.g., *17).

**FIGURE 1:**
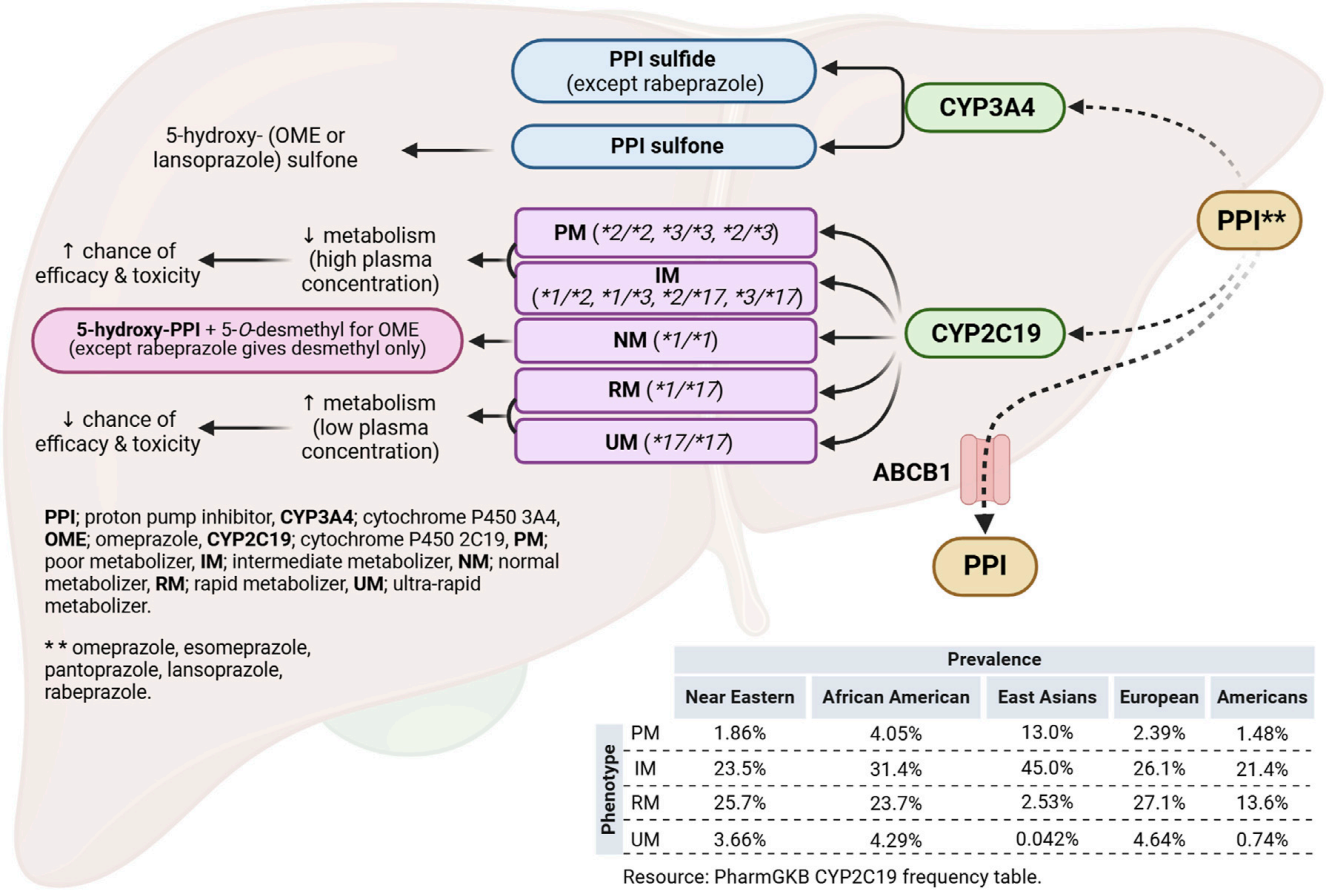
Legend. Hepatic metabolic pathways of proton pump inhibitors and the effect of *CYP2C19* polymorphism. Created using Biorender.com. Adel, A. (2023) BioRender.com/e86w246.

Based on the *CYP2C19* allele pair (genotype), the phenotypes of individuals could be classified as outlined by the Clinical Pharmacogenetics Implementation Consortium (CPIC) into: poor metabolizers (PMs), intermediate metabolizers (IMs), normal metabolizers (NMs), rapid metabolizers (RMs), or ultra-rapid metabolizers (UMs) (Lam et al., 2019) (Figure 1).

According to the pharmacogenomics knowledge base (PharmGKB), Oceanian population (60.95%) has the highest frequency for the *2 allele followed by East Asians/HAN Chinese (30.35%), Central/South Asians (26.99%), African American/Afro-Caribbeans (18.15%), and Europeans (14.69%). Similarly, *3 allele is most prevalent among Oceanians (14.64%) and East Asians/HAN Chinese (6.35%). In contrast, *17 allele frequency is reported to be highest in Europeans (21.54%) and African Americans/Afro-Caribbeans (20.72%) and lowest in Oceanians (5.7%) and East Asians (2.05%). For the phenotypes, East Asians/HAN Chinese population has the highest frequency (13.0%) for the PM phenotype followed by African Americans (4.05%), European (2.39%), Near Eastern (1.86%), and Americans (1.48%). Furthermore, the frequency of the IM phenotype is highest in East Asians/HAN Chinese population (34%), followed by African Americans (31.4%), European (26.1%), and Near Eastern (23.5%) and lowest in Americans (21.4%). In contrast, the European population is reported to have the highest frequency for the RM (27.1%),followed by Near Eastern [25.7%]) and African Americans (23.7%) and (Figure 1) (Whirl-Carrillo RH et al., 2021).

The effect of *CYP2C19* polymorphism on each PPI varies, with omeprazole being the most affected followed by lansoprazole, dexlansoprazole, pantoprazole, esomeprazole, and rabeprazole (Eken et al., 2023). The *CYP2C19* genotype and phenotype are important predictors of PPIs therapeutic response and toxicity. For instance, RMs (e.g., Europeans, Near Eastern) and UMs (e.g., Europeans, African Americans) may experience therapeutic failures due to increased metabolic inactivation, while IMs (e.g., African Americans, Europeans) and PMs (e.g., East Asians, African Americans) may experience enhanced therapeutic effects but with greater safety concerns and PPIs-related side effects (Chevalier et al., 2023).

Emerging evidence suggest an association between *CYP2C19* polymorphisms, PPIs plasma concentrations, and PPIs-related adverse drug reactions (ADRs) (Cicali et al., 2023). Due to this concern, genotype-guided dosing of PPIs has been recommended by the CPIC and the Dutch Pharmacogenetics Working Group (DPWG). The CPIC guidelines suggest that IMs and PMs are at a higher risk of ADRs and recommend dose reduction for these individuals. In contrast, the DPWG guidelines recommend no dose adjustment in these phenotypes, noting that an increased plasma exposure does not correlate with an increase in PPIs-related ADRs. Overall, a consensus on genotype-guided dosing recommendation is not yet well-established due to the paucity of a robust evidence (Whirl-Carrillo RH et al., 2021).

The incidence of PPIs-related ADRs, particularly with prolonged use, has been reported in several studies (Haastrup et al., 2018). However, evidence regarding the long-term PPIs-related ADRs and their association with *CYP2C19* genetic variants is limited. Therefore, the objective of this narrative review was to provide an overview of long-term ADRs associated with PPIs, and to review the current evidence linking *CYP2C19* genotypes to these PPIs-related ADRs.

## 2 PPIs-related ADRs and association with *CYP2C19*

The long-term use of PPIs is clinically described as treatment lasting more than 4 weeks for ulcers and more than 8 weeks for reflux symptoms (Haastrup et al., 2021). PPI-induced ADRs are diverse, affecting several body systems, including hematological, renal, musculoskeletal, gastrointestinal (GI), respiratory, and the central nervous systems.

### 2.1 Electrolytes imbalance

#### 2.1.1 Hypomagnesemia

The association between magnesium level and long-term PPIs use has been assessed in many observational studies. The proposed mechanism by which PPIs induce hypomagnesemia is through inhibition of the transient receptor protein channels (TRPM 6/7) responsible for the active intestinal absorption of magnesium (Abdelwahab and John, 2023). Studies have shown conflicting findings; while some studies reported hypomagnesemia associated with PPIs use (Zipursky et al., 2014; Cheungpasitporn et al., 2015), others found no significant risk (Bahtiri et al., 2017). However, the change in magnesium levels reported in the studies may be attributed to factors such as concomitant medications taken with the PPIs (e.g., diuretics), study heterogeneity, or confounding variables (Haastrup et al., 2018). Although increased PPIs exposure in *CYP2C19* PMs and IMs is suggested to be the cause of electrolyte imbalance (El et al., 2018), none of the studies investigated the effect of *CYP2C19* variants on the occurrence of ADRs related to PPIs use. Hence, it remains unclear whether PPIs-related hypomagnesemia is associated with *CYP2C19* genetic predisposition or not.

#### 2.1.2 Hyponatremia

PPIs-related hyponatremia is a rarely reported complication in published literature, and there is currently no definitive association established between PPIs and hyponatremia. It is hypothesized that PPIs may disrupt the production of antidiuretic hormone (ADH), leading to fluid retention and subsequent decrease in sodium level. Evidence suggest that discontinuation of PPIs can correct hyponatremia, while re-challenge may induce hyponatremia again, suggesting a potential link between PPIs use and imbalance in sodium level (Brewster and Perazella, 2007; Mennecier et al., 2005; Marcianò et al., 2024). Similar to hypomagnesemia, increased exposure to PPIs with some *CYP2C19* phenotypes is the proposed mechanism (El et al., 2018). Nevertheless, the role of underlying *CYP2C19* genetic mutations in the development of hyponatremia associated with PPIs is yet to be investigated, leaving a gap in understanding the genetic association of this ADR.

#### 2.1.3 Hypocalcemia

The mechanism by which PPIs suppress gastric acid production is thought to affect the proper absorption of calcium. Changes in calcium level in PPIs users have been discussed in a few case reports and prospective comparative studies. For instance, two case reports indicated the normalization of calcium level after PPIs discontinuation, without the need for supplementation (Epstein et al., 2006). Furthermore, a 12-month comparative study found a significant decrease in total serum calcium and parathyroid hormone (PTH) concentration, but concluded that further evidence is needed before making a clinical recommendation for monitoring or supplementation of calcium levels (Bahtiri et al., 2017).

Similar to magnesium and sodium, the current literature review showed that there is no well-established association between hypocalcemia, PPIs use, and *CYP2C19* genetic variants. However, it is plausible that increased exposure to PPIs in individuals who are PMs and IMs could cause further reduction in electrolyte levels, leading to imbalances (El et al., 2018).

### 2.2 Kidney complications

Acute interstitial nephritis (AIN) is a possible cause of acute kidney injury (AKI) that can lead to life-threatening deterioration in kidney function. Several reports have described cases of AIN associated with PPIs use, and it is considered a class effect of PPIs. While renal function is usually restored in many cases, some degree of deterioration in kidney function may persist even after stopping the medication (Brewster and Perazella, 2007). This highlights the need for consideration of potential renal complications associated with long-term PPIs use. Two cases of AKI have been reported and linked to genetic polymorphisms, including *CYP2C19* no-function allele *2. However, no definitive conclusion could be drawn due to other confounding factors such as polypharmacy and potential drug-drug interactions (DDIs) (Leung et al., 2009). In another study, 20 patients with omeprazole-induced AIN were genotyped to determine the presence of *CYP2C19* no-function alleles (particularly *2 and *3). This research aimed to clarify the role of genetic factors in the development of AIN associated with PPIs use, but further investigation is needed to establish clear links. Although one-third of patients were found to have the no-function allele, no significant association was established between this genetic variant and the development of AIN. This finding was attributed to the small sample size of the study and to the elderly population included, who are known to have reduced metabolic function, which further predisposes them to ADRs (Helsby et al., 2010). These factors suggest that larger studies with more diverse populations may be necessary to better understand the relationship between *CYP2C19* polymorphisms and AIN.

### 2.3 Galactorrhea

Galactorrhea is associated with estrogen or testosterone deficiency in both females and males. Currently, only case reports are available regarding PPIs-related galactorrhea. The first case report described an increased metabolism of testosterone in a CYP2C19*2 female patient receiving esomeprazole (Rosenshein et al., 2004). It was suggested that the high levels of esomeprazole, resulting from decreased clearance, might have induced the metabolism of testosterone, leading to loss of libido and other complications (Rosenshein et al., 2004). The second case report involved a female kidney transplant recipient on a high dose of omeprazole who subsequently developed sudden galactorrhea and hyperprolactinemia. These side effects were determined to be associated with omeprazole, although no investigations were conducted on the *CYP2C19* variants (Prikis et al., 2020). The third case reported symptoms of galactorrhea that developed 3 days after initiation of omeprazole in a female patient. The causal relationship between omeprazole and galactorrhea was confirmed through discontinuation and re-challenge (Dorji et al., 2022). Based on this, disturbance to the levels of sex hormones, and consequently galactorrhea, is more likely to occur in individuals with increased CYP2C19 activity. These cases highlight the potential for PPIs to induce hormonal changes, warranting further research into the mechanisms involved and the role of *CYP2C19* polymorphisms.

### 2.4 Bone complications

PPI-associated bone complications may be linked to their effect on calcium and PTH levels. The short-term use of PPIs is believed to increase the risk of vertebral, wrist, and hip fractures, by causing a disturbance in bone absorption-resorption balance (Laria et al., 2011). A retrospective cohort study found that PMs are not associated with any increased risk for fracture. However, there are some concerns related to the study power and participants heterogeneity that limit the ability to establish a definitive association (Gronich et al., 2022). A recent hospital-based study investigated the long-term effects of PPIs on fracture risk and bone mineral density (BMD), as well as their association with *CYP2C19* genetic variants (*2, *3, *6) This study found no significant associations between IM and NM (Hazard ratio [HR], 0.93; 95% CI, 0.52–1.69; *p* = 0.822) or between PM and NM (HR, 0.64; 95% CI, 0.24–1.72; *p* = 0.378), suggesting that the findings require further investigation. (Liao et al., 2023).

### 2.5 Hypergastrinemia

The reduction in gastric acidity caused by proton pump inhibition triggers the secretion of gastrin, a hormone that increases acid production. In some cases, this increase is reported to cause hypergastrinemia (Festen et al., 1984; Shiotani et al., 2018). The relationship between long-term omeprazole use (>1 year), *CYP2C19* genetic variants, and gastrin level was investigated in 180 patients with acid-related disorders. The findings revealed that patients with one or two no-function alleles (*2 and/or *3) showed higher serum levels of gastrin and chromogranin A compared to those with normal-function alleles (*p* = 0.0001) (Sagar et al., 2000). This finding suggests that genetic variations in *CYP2C19* may play a role in PPI’s effect on gastrin levels.

### 2.6 Infections

#### 2.6.1 *Clostridium Difficile* infection (CDI)

*Clostridium Difficile* (CD) is an anaerobic, Gram-positive, toxin-producing bacteria that can cause symptoms ranging from diarrhea to life-threatening complications. The acidic gastric environment is negatively affected by chronic use of PPIs. Consequently, the natural defense mechanism in the stomach is weakened, making it more susceptible to microbial invasion. Several observational studies have reported high incidence and recurrence of *Clostridium difficile* infection (CDI) with prolonged PPIs use (Laria et al., 2011). Moreover, lower GI infection rates were observed in pediatric patients with high CYP2C19 activity (one and/or two *17), due to lower exposure to PPIs compared to normal CYP2C19 activity (Bernal et al., 2019). However, this may also indicate decreased therapeutic efficacy of PPIs, which may possibly lead to therapeutic failure.

#### 2.6.2 Respiratory tract infection (RTI)

Upper RTIs (URTIs) and lower RTIs (LRTIs) such as rhinitis, pharyngitis, and pneumonia may be triggered by PPIs-related hypochlorhydria. This is hypothesized to be caused by the higher bacterial content of the micro-aspirate coming from the gastric region, gastric acid level is low (Haastrup et al., 2018). Patients with less exposure to PPIs, such as RMs/UMs, are reported to have lower rate of infections. In a cohort study of 670 children, it was observed that the infection rate was lower in RMs/UMs (one and/or two *17) versus *CYP2C19* NMs [odds ratio (OR), 0.7; 95% CI, 0.50–0.97; *p* = 0.03], but no significant difference was observed when compared to PMs or IMs (*p* = 0.1), (Bernal et al., 2019). In another study involving 306 children using lansoprazole, the PMs phenotype (*2, *3, *8, *9 alleles) was reported to have a higher frequency of upper RTIs (OR, 2.46; 95% CI, 1.02–5.96; *p* = 0.046) compared to the EMs phenotype (one and/or two *17 alleles). In addition, there was a significant difference in the incidence of URTI between EMs and the placebo group (not taking lansoprazole), with EMs having a higher incidence (*p* = 0.0039) (Lima et al., 2013).

### 2.7 Asthma

The relationship between *CYP2C19* and asthma control is linked to the high risk of respiratory infections that results from hypochlorhydria developed from PPIs use. In a double blinded trial involving 306 children with asthma, the group given lansoprazole and were PMs (*2, *3, *8, *9, *10) experienced worse asthma control due to higher incidence of RTI at 6 months in comparison with EMs (*p* = 0.0039) (Lima et al., 2013; Lang et al., 2015). Based on these findings, other studies suggest the potential benefits of genotype-guided dosing of PPIs for the treatment of acid-related disorders in patients with uncontrolled asthma (Tang et al., 2019).

### 2.8 Migraine

The prevalence of headaches and migraines associated with PPIs use has been evaluated in multiple studies. While it has been shown that PPIs are associated with a higher risk of headaches and migraines, the specific association with *CYP2C19* is still unclear. It is suggested that prolonged exposure to PPIs might have an impact on the brain in some patients, but the exact mechanism is still unknown (Liang et al., 2015; Makunts et al., 2019). One study investigating the association between migraine incidence and *CYP2C19* phenotypes stated that male patients with decreased CYP2C19 activity (*2, *3, *8, *9) are at a higher risk of experiencing PPIs-related migraine (OR, 2.11; 95% CI, 1.04–4.29; *p* = 0.038) compared to men with other phenotypes and females (Pisanu et al., 2021). These indicate the need for further research to better understand the relationships between genetic factors, PPIs use, and migraine susceptibility.

A summary of PPIs-related ADRs, *CYP2C19* variants involved, and the plausible mechanism is provided in Table 1.

**TABLE 1:** A summary of PPIs-related ADRs, *CYP2C19* variants involved, and the plausible mechanism.

ADR	ADR description	Associated PPI	*CYP2C19* genetic alleles involved	Plausible mechanism
Hypomagnesemia	Low serum magnesium level	Not specified	PMs and IMs without specification of alleles	Affecting the transient receptor protein channels (TRPM 6/7) responsible for the active intestinal absorption of magnesium
Hyponatremia	Low serum sodium level	Not specified	PMs and IMs without specification of alleles	Disturb the production of antidiuretic hormone (ADH) leading to fluid retention
Hypocalcemia	Low total serum calcium and parathyroid hormone (PTH)	Not specified	PMs and IMs without specification of alleles	Suppressing gastric acid is thought to affect the proper absorption of calcium
Acute Interstitial Nephritis (AIN)	Deterioration in renal function that is sustained after stopping PPIs	Omeprazole	*2, *3	NA
Galactorrhea	Estrogen or testosterone deficiency leading to galactorrhea	Esomeprazole, omeprazole	*2	Increased metabolism of estrogen or testosterone
Bone Complications	Osteopenia, osteoporosis, fracture, low BMD	Not specified	*2, *3, *6	Could be linked to the effect on calcium and PTH levels
Hypergastrinemia	Low serum gastrin and chromogranin A	Not specified	*2, *3	Reduction in acidity caused by PPIs inhibition of proton pump triggers the secretion of gastrin
*Clostridium Difficile* Infection (CDI)	Range of symptoms from diarrhea to life-threatening damage	Not specified	*1, *17	Natural body defense in the stomach is weakened as the gastric environment is negatively affected
Respiratory Tract Infection (RTI)	Upper and lower RTIs such as rhinitis, pharyngitis, and pneumonia	Lansoprazole	*1, *2, *3, *8, *9	Could be caused by the high bacterial content of the micro-aspirate coming from the gastric region that have low availability of gastric acid
Asthma	Uncontrolled asthma, exacerbation	Lansoprazole	*2, *3, *8, *9, *10	Related to the high risk of infections
Migraine	Headache, migraine	Not specified	*2, *3, *8, *9	Prolonged exposure to PPIs in some patients might have an impact on the brain

## 3 Discussion

This review outlined PPIs-related long-term ADRs and summarized the existing evidence on their association with *CYP2C19* polymorphic alleles. Future research should investigate the association between long-term PPIs-related ADRs and *CYP2C19* phenotypes using more robust study designs. This would be an essential step towards advocating for a genotype-guided dosing of PPIs as a measure to enhance the safe and effective prescribing of PPIs, especially for older adults, and during long-term use. This strategy may help mitigate the risks of PPIs-related ADRs while optimizing therapeutic outcomes.

## 4 Conclusion

This review of current literature regarding *CYP2C19* genotypes and PPIs-related ADRs suggests that while there are established associations between PPIs and their ADRs, evidence linking these reactions to *CYP2C19* variants remains limited. The role of *CYP2C19* in the development of long-term ADRs associated with PPIs is of increasing interest. Therefore, larger cohorts with robust study design are essential to provide a more robust assessment of these relationships in clinical practice.
